# Association of single nucleotide polymorphisms in *Neuropeptide Y (NPY)* and *Phosphoglycerate Mutase 2 (PGAM2)* genes with growth traits in rabbits

**DOI:** 10.1007/s11250-024-04085-w

**Published:** 2024-08-12

**Authors:** Mostafa Helal, Marwa Ahmed, Mohamed Ragab, Ahmed Ateya, Shimaa Sakr

**Affiliations:** 1 Department of Animal Production, Faculty of Agriculture, Cairo University, Giza, 12613 Egypt; 2 Department of Animal Production, National Research Centre, Dokki, Giza, Egypt; 3 Poultry Production Department, Faculty of Agriculture, Kafrelsheikh University, Kafrelsheikh, Egypt; 4 Animal Breeding and Genetics Department, National Institute for Agricultural and Food Research and Technology (INIA), Madrid, 28040 Spain; 5 Department of Development of Animal Wealth, Faculty of Veterinary Medicine, Mansoura University, Mansoura, 35516 Egypt

**Keywords:** Candidate genes, Growth, NPY, PGAM2, Rabbits, SNP

## Abstract

Genetic improvement of local rabbit breeds using modern approaches such as marker-assisted selection requires accurate and precise information about marker‒trait associations in animals with different genetic backgrounds. Therefore, this study was designed to estimate the association between two mutations located in the *Neuropeptide Y* (*NPY*, g.1778G > C) and *Phosphoglycerate Mutase 2* (*PGAM2*, c.195 C > T) genes in New Zealand White (NZW), Baladi (BR), and V-line rabbits. The first mutation was genotyped using high-resolution melting, and the second mutation was genotyped using the PCR-RFLP method. The results revealed significant associations between the *NPY* mutation and body weight at 10 (V-line) and 12 weeks of age (NZW, BR, and V-line), body weight gain (BWG) from 10 to 12 weeks of age (BR), BWG from 6 to 12 weeks of age (NZW, BR, and V-line), average daily gain (NZW, BR, and V-line, and BR), growth rate (GR) from 8 to10 weeks (V-line), 10 to 12 weeks (BR), and GR from 6 to 12 weeks of age (BR, and V-line). The *PGAM2* mutation was associated with body weight at 10 (V-line) and 12 (NZW, and V-line) weeks of age, with significant positive additive effects at 12 weeks of age in all breeds, and was associated with BWG from 8 to 10 and 10 to 12 in BR, and BWG from 6 to 12 weeks of age (NZW, and BR), and average daily gain (NZW, and BR), and was associated with GR form 8 to 10 weeks (BR), from10 to 12 weeks (BR, and V-line) and from 6 to 12 weeks (BR). The results highlighted the importance of the two mutations in growth development, and the possibility of considering them as candidate genes for late growth in rabbits.

## Introduction

Breeding practices and domestication have impacted growth performance (El-Sabrout and Soliman 2018). Alves et al. (2015) examined the effects of domestication on the genetic diversity of rabbits and discovered evidence for two significant genetic diversity reductions, one of which was linked to breed creation. Due to feeding costs and consumer preferences for lean meat, selection based on growth characteristics has been extremely important to the rabbit business (El-Sabrout and Soliman 2018). Body weight gain is one of the most economically important traits in rabbits (Cartuche et al. 2014), and it has been a selection criterion in different selection programs (Mínguez et al. 2016). However, traditional rabbit breeding is challenged by many factors, including the poor data and information about relationships between relatives, the limited number of animals, and the sensitivity of rabbits to high-temperature weather (Khalil 2020; Sabry et al. 2021).

Marker-assisted selection (MAS) is a powerful tool in animal breeding programs to improve the efficiency and accuracy of selection (ABDEL-KAFY et al. 2016). Additionally, in MAS, genetic markers such as microsatellites and single-nucleotide polymorphisms (SNPs) are considered as bioinformatics indicators to provide detailed information regarding polymorphism in allelic genotypes at a certain locus (Bovo et al. 2021). This information enables in-depth analyses and evaluations of genetic variants as well as the identification of genes impacting economically significant features in farm animals (Carneiro et al. 2011). Single-nucleotide polymorphisms of numerous candidate genes have been used to identify genes related to body weight and meat quality in rabbits (Fontanesi et al. 2006; Wu et al. 2015; Helal et al. 2022). According to Seidel (2010), using SNP markers in genomic selection is a powerful new tool, because SNPs can be detected using a variety of simple techniques, such as PCR-RFLP, and therefore SNPs can be used for mass screening of many samples in a short time (Fontanesi et al. 2006). Moreover, SNPs are the most recent contribution to the study of DNA sequence variation, and SNPs represent the most innovative molecular marker in genotyping studies.

The complex neuroendocrine system that regulates energy balance includes both central and peripheral signals, including neuropeptides (Hillebrand et al. 2002). Feeding-related neuropeptides are a part of this intricate control system, and orexigenic neuropeptides such as Neuropeptide Y (NPY) are crucial for controlling energy balance and feed intake (Bazhan and Zelena 2013). The 36 amino acid neuropeptide NPY was initially identified in the pig brain in 1982 and is a plentiful and well-characterized neuropeptide (Mercer et al. 2011). The stimulation of food intake and correlation with weight gain in mammals are the strongest effects of this powerful orexigenic factor (Hillebrand et al. 2002; Ding et al. 2005). In animals, intracerebroventricular (ICV) NPY injection boosted appetite in a dose-dependent manner (Clark et al. 1997). As feed intake increases, NPY eventually decreases after initially increasing just before the start of eating (Kalra et al. 1991). In animal obesity models, tubby mice and rats without brown adipose tissue showed overexpression of the NPY gene (Guan et al. 1998). Additionally, it has been discovered that the bovine *NPY* gene is positioned in the QTL region for carcass characteristics, marbling, and lean meat (Casas et al. 2004; Mizoshita et al. 2004). In rabbits, SNPs in the *NPY* gene were significantly associated with eviscerated slaughter percentage and semi-eviscerated slaughter percentage in rabbits (Liu et al. 2014).

Phosphoglycerate mutase (PGAM) is an enzyme in the glycolytic pathway that converts 3-phosphoglycerate into 2-phosphoglycerate to release energy during glycolysis in muscle tissues (Fontanesi et al. 2006). The PGAM dimer in mammalian tissues is composed of the brain form (B form, also known as PGAM1), which is extensively expressed, and the muscle form (M form, also known as PGAM2), which is exclusively present in adult skeletal and cardiac muscle. According to Qiu et al. (2008), PGAM2 is connected to growth, feed conversion, and slaughter quality and is highly expressed in skeletal muscle during all periods of pig development. Additionally, *PGAM2* plays crucial functions in the glycolysis process that regulates postnatal growth and associated meat quality indices (Sevane et al. 2013).

To our knowledge, there is little information on the relationship between growth traits in domestic rabbit breeds and genetic variations in *Neuropeptide Y* (*NPY*) and *Phosphoglycerate Mutase 2* (*PGAM2*) genes. Therefore, the goal of this work was to determine the association between growth features in the New Zealand White (NZW), Valencian line V (V-line), and Baladi rabbit (BR) breeds and single nucleotide polymorphisms in the *NPY* and *PGAM2* genes.

## Materials and methods

### Animals and management

Three rabbit breeds were used for the current study, including two commercial lines and one local breed. The commercial lines were New Zealand White (NZW) rabbits, which is a commercial rabbit breed that originated and developed in the USA and was raised in Egypt at the commercial level for many decades. The other commercial line was the Valencian line V (V-line), which was developed in Spain, and has been selected for litter size at weaning (Ragab and Baselga 2011), and then imported and bred in Egypt in the past few decades. Concerning the local breed, improved Baladi rabbit Breeds (BR), which were developed by crossbreeding for several generations between native Egyptian rabbits and Flemish Giant (Khalil 1999). The number of rabbits was 92, 60, and 76 for NZW (40 males and 52 females), BR (26 males and 34 females), and V-line rabbits (33 and 43 individuals), respectively.

The kits of the different genetic groups were weaned at the same age (35 ± 1 d). Rabbits were collected from different farms in Giza, Fayoum, and Zagazig governorates in Egypt, and all rabbits were genetically unrelated. After receiving rabbits, they were kept for one week for an adaptation period. The rabbits were housed in the same room and received the same management and environmental conditions. The housing system was a semi-open housing system including cages (60 × 50 × 40 cm) supplied with a nipple system and feeding hoppers. A commercial fattening diet (18% protein, 2750 ME, and 12% crude fiber) was provided for all rabbits, and water was provided *ad libitum*. At the end of the experimental procedures animals were released.

Individual body weights (BW, g) were recorded every two weeks until marketing age (From 6 to 12 weeks of age), and body weight gains for different periods (BWG, g) were calculated as the difference between body weight at the beginning and end of the period. Relative growth rates (GR, %) were calculated as BWG for a certain period divided by the average BW at the start and end of the period.

### Samples and genotyping

Blood samples were collected from all rabbits at the end of the fattening period. Samples were collected in tubes containing ethylene diamine tetra-acetic acid (EDTA) and stored at -20 °C until use. DNA was extracted using a whole genomic extraction kit (Wiz-bio-solution Inc., South Korea). Two SNPs were genotyped. The first SNP (g.1778G > C), located in the intron 1 of the *NPY* gene, was genotyped using high-resolution melting (HRM). Primers (Table 1) were designed to amplify a fragment of 98 bp containing the SNP, which was also described previously (Liu et al. 2014). The HRM analysis was performed in a total volume of 10 µl using HOT FIREPol EvaGreen HRM Mix (Solis BioDyne, Estonia). The reaction mixture included 5 µl of EvaGreen HRM mix, 1 µl of DNA template, 0.5 µl of each primer, and 3 µl of ddH2O. The HRM reaction profile included initial denaturation at 95 °C for 12 min to activate HOT FIREPOL DNA polymerase; followed by 40 cycles of 95 °C for 20 s, 48 °C for 20 s, and 72 °C for 20 s; and an HRM step from 65 to 95 °C rising at 0.4 °C.

**Table 1:** Primer sequences and genotyping methods for the genotyped SNPs in the NPY and PGAM2 genes

Gene	SNP	Genotyping method	Primer sequences (5’- 3’)	Annealing	Reference
*NPY*	g.1778G > C	HRM	F: ACGTTGGATGTCTAAACTGGGTTGGAAGCG	48 °C	(Liu et al. 2014)
			R: ACGTTGGATGTCTAAACTGGGTTGGAAGCG		
*PGAM2*	c.195 C > T	PCR-RFLP using Csp6I	F: GAATGCTGATTGGCAGTTGGC	62 °C	(Wu et al. 2015)
			R: CCAGTTGTCTGAAACCCCTGTG		

The second mutation was the c.195 C > T SNP of the *PGAM2* gene, which was genotyped using restriction fragment length polymorphism-polymerase chain reaction (RFLP-PCR) analysis as described previously (Wu et al. 2015). The primers were used to amplify 855 bp of the 5´ UTR to intron 2 of the *PGAM2* gene (Table 1). PCR was performed in a total reaction volume of 20 µl, containing 1 µl of each of the two primers, 2 µl of DNA template, 10 µl of PCR master mix, and 6 µl of PCR grade water. The reaction was carried out with an initial denaturation cycle at 95 °C for 5 min, 34 cycles of 95 °C for 40 s, 62 °C for 40 s, and 72 °C for 40 s, and an additional 10 min at 72 °C as an extension cycle. The PCR products (10 µl) were then incubated with 10 U of *Csp6I* restriction enzyme (Vivantis, Malaysia) at 37 °C, followed by electrophoresis on a 2.5% agarose gel.

### Statistical analysis

Genetic parameters including heterozygosity (He), effective number of alleles (Ne) and polymorphic information content (PIC) were calculated according to Botstein et al. (Botstein et al. 1980). The association analysis was performed using the general linear model (GLM) of SAS (SAS 1999), using the following model:

$${Y_{ijklm}}\; = \;\mu \; + \;{G_i}\; + \;{S_j}\; + {B_k}\; \cdot \;{R_l}\; + \;{\varepsilon _{ijklm}}$$

where *Y*_*ijk*_ is the trait recorded of animal *l*^*th*^, *µ* is the overall mean of trait, G*i* is the genotype effect (three levels; p_2_, 2pq, and q_2_ ), *S*_*j*_ is the sex effect (two levels; male and female), *B*_*k*_ is the breed effect (three levels; NZW, BR, and V-line), *R*_*l*_ is the source of rabbits (three levels; farm A, farm B, and farm C), and *ε*_*ijkl*_ is the random residual of the model.

## Results

### SNP identification

The c.195 C > T SNP was detected via PCR-Csp6I digestion of 855 kb of the *PGAM2* gene. Table 2 showed the allelic and genotypic frequencies, as well as population genetic parameters, including He, PIC, and Ne. The three genotypes were found in the NZW, V-line, and BR breeds, with frequencies of 0.50, 0.42, and 0.61 for the heterozygous genotype (CT) being predominant in each breed. The c.195 C > T SNP locus was classified by PIC as having moderate genetic diversity, which suggested that this SNP has a sizable amount of selection potential. Additionally, for this SNP, no rabbit breeds showed any deviation from HWE.

The three genotypes of the *NPY* gene were detected by HRM g.1778G > C SNP analysis (Table 2). In NZW and V-line rabbits, the heterozygous genotype (CG) predominated with frequencies of 0.41 and 0.47, respectively. However, with a frequency of 0.47, the homozygous genotype AA predominated in BR rabbits. The g.1778G > C SNP locus was classified by PIC as having considerable genetic diversity, which suggested that this SNP had a sizable selection potential. Similarly, for this SNP, no rabbit breeds showed deviation from HWE.

**Table 2:** Number of genotyped animals, genotypic frequency, allelic frequency and deviation from Hardy Weinberg of the NPY, and PGAM2 loci

Breed	Number of animals							Genotypic frequency					Allelic frequency				Genetic parameters				Deviation form HW		
								p2	2pq	q2			*p*		q		He	Ne	PIC		χ^2^	*p* value	
	***NPY (*** **g.1778G > C** ***)***																						
	*CC*		*CG*		*GG*																		
NWZ	24		38		30			0.26	0.41	0.33			0.47		0.53		0.498	2.007	0.374		2.670		0.102
BR	28		22		10			0.47	0.37	0.17			0.65		0.35		0.455	2.198	0.352		2.260		0.130
V-line	18		36		22			0.24	0.47	0.29			0.47		0.53		0.498	2.007	0.374		0.190		0.662
	***PGAM2 (*** **c.195 C > T** ***)***																						
*CC*	*CT*		*TT*																				
NWZ	22		46		24			0.24	0.50	0.26			0.49		0.51		0.500	2.001	0.375		2.056		0.996
BR	24		25		11			0.40	0.42	0.18			0.61		0.39		0.476	2.102	0.363		0.9468		0.330
V-line	14		46		16			0.18	0.61	0.21			0.49		0.51		0.500	2.001	0.375		3.395		0.065
.																							

NWZ = New Zealand rabbits, BR = Baladi rabbits, He = expected heterozygosity, Ne = number of effective alleles, PIC = polymorphic information content

χ2 represents the balance of Hardy–Weinberg of different genotypic distributions: χ^2^ 0.05 = 3.84, χ^2^ 0.01 = 6.63

### Association of NPY and PGAM2 genes with growth traits in rabbits

The SNP g.1778G > C of the *NPY* gene has impacts on growth features in all breeds, as evidenced by the data in Table 3. In NZW rabbits, individuals with the G allele had higher body weight at 12 weeks of age (BW12), body weight gain from 10 to 12 weeks of age (BWG1012), body weight gain from 6 to 12 weeks of age (BWG612), and average daily gain (ADG) than those with the CC genotype. The additive effect of the SNP was significant for BW12, BWG810, and BWG612; however, the dominance effect was significant for BW12, and BWG612. For the BR rabbits, the GG and CG genotypes had higher BW12, BWG1012, BWG612, ADG, GR1012, and GR612 than the CC genotype. The additive and dominance effects of the SNP were significant for BW12, BW1012, BW612 and GR612. Meanwhile, the GG and GC rabbits exhibited higher BW10, BW12, BWG612, ADG, GR810 and GR612 than the CC genotype for the V-line breed. However, there was no discernible difference in the different traits between GG and CG individuals. The additive effect was significant for BW10, BW12, BW1012 and BWG612, and the dominance effect was significant for BW10, and BW12.

**Table 3:** Association between genotypes of the g.1778G > C mutation of the *NPY* gene and growth traits (Mean ± SD) in different rabbit breeds

	Genotype			*p*-value	Additive	Dominance
Trait	CC	CG	GG			
	**New Zealand White rabbits**					
BW6	830.92 ± 109.84	848.27 ± 87.94	860.77 ± 61.99	0.4597	-27.56	10.57
BW8	1215.83 ± 92.13	1237.61 ± 107.30	1242.73 ± 72.64	0.5433	9.45	17.90
BW10	1816.96 ± 113.46	1851.18 ± 115.87	1860.53 ± 80.85	0.2920	66.78	7.67
BW12	2260.25^b^ ± 118.27	2358.18^a^ ± 124.21	2380.77^a^ ± 121.90	0.0012	77.44**	35.90*
BWG68	384.92 ± 88.31	389.34 ± 88.67	381.97 ± 75.03	0.9364	37.01	7.32
BWG810	601.13 ± 107.76	613.58 ± 119.60	617.80 ± 101.68	0.8526	57.33*	-10.24
BWG1012	443.29^b^ ± 105.31	507.00^a^ ± 97.20	520.23^a^ ± 123.38	0.0267	10.65	28.24
BWG612	1429.33^b^ ± 130.29	1509.92^a^ ± 132.32	1519.00^a^ ± 120.37	0.0224	105.00**	25.33**
ADG	34.03^b^ ± 3.10	35.95^a^ ± 3.15	36.19^a^ ± 2.87	0.0225	2.50	0.60
GR68	0.095 ± 0.025	0.093 ± 0.022	0.092 ± 0.018	0.8189	0.06	0.00
GR810	0.098 ± 0.018	0.100 ± 0.021	0.099 ± 0.017	0.9507	0.04	-0.02
GR1012	0.054 ± 0.013	0.060 ± 0.012	0.062 ± 0.014	0.0916	0.00	0.02
GR612	0.233 ± 0.025	0.236 ± 0.020	0.235 ± 0.016	0.8521	0.18	0.01
	**Baladi Rabbits**					
BW6	658.11 ± 90.35	632.45 ± 78.54	669.7 ± 39.03	0.825	-16.71	-0.23
BW8	909.57 ± 88.77	901.68 ± 111.68	882.7 ± 76.96	0.511	-57.40*	17.20
BW10	1501.64 ± 176.96	1403.86 ± 156.2	1471.6 ± 190.47	0.1425	47.28	-50.76
BW12	1839.25^b^ ± 180.86	2017.55^a^ ± 242.6	2023.50^a^ ± 101.53	0.0035	79.05**	122.38***
BWG68	251.46 ± 98.22	269.23 ± 99.56	213 ± 65.26	0.3023	-40.69	17.44
BWG810	592.07 ± 157.4	502.18 ± 104.58	588.9 ± 170.08	0.0745	104.68	-67.96
BWG1012	337.61^b^ ± 96.13	613.68^a^ ± 152.33	551.90^a^ ± 174.74	0.0001	31.76**	173.13**
BWG612	1181.14^b^ ± 148.41	1385.09^a^ ± 210.93	1353.80^a^ ± 77.23	0.0002	95.75*	122.61*
ADG	28.12^b^ ± 3.53	32.98^a^ ± 5.02	32.23^a^ ± 1.84	0.0002	2.28	2.92
GR68	0.081 ± 0.033	0.086 ± 0.031	0.069 ± 0.019	0.3453	-0.06	0.02
GR810	0.122 ± 0.028	0.11 ± 0.021	0.123 ± 0.027	0.2370	0.18	-0.10
GR1012	0.051^b^ ± 0.015	0.089^a^ ± 0.021	0.079^a^ ± 0.029	0.0001	0.02	0.13
GR612	0.236^b^ ± 0.022	0.261^a^ ± 0.023	0.251^a^ ± 0.009	0.0002	0.19*	0.19*
	**V-line Rabbits**					
BW6	977.11 ± 123.75	939.11 ± 79.97	964.45 ± 77.08	0.3103	43.42	4.06
BW8	1299.44 ± 81.52	1316.11 ± 101.25	1346.91 ± 115.78	0.3184	41.45	30.36
BW10	1760.44^b^ ± 141.01	1888.44^a^ ± 134.11	1895.55^a^ ± 154.61	0.0044	46.39*	109.91**
BW12	2288.44^b^ ± 96.05	2391.11^a^ ± 99.18	2352.00^a^ ± 122.07	0.0049	89.27*	64.08*
BWG68	322.33 ± 101.25	377.00 ± 84.35	382.45 ± 99.31	0.0824	-1.96	26.31
BWG810	461.00 ± 99.02	572.33 ± 123.84	548.64 ± 142.64	0.0824	4.95	79.53
BWG1012	528.00 ± 108.57	502.67 ± 105.29	456.45 ± 105.10	0.0960	42.88*	-45.83
BWG612	1311.33^b^ ± 112.57	1452.00^a^ ± 109.22	1387.55^a^ ± 112.18	0.0002	45.86*	60.02
ADG	31.22^b^ ± 2.68	34.57^a^ ± 2.60	33.04^a^ ± 2.67	0.0002	1.09	1.43
GR68	0.071 ± 0.025	0.084 ± 0.018	0.083 ± 0.019	0.0720	-0.01	0.03
GR810	0.074^b^ ± 0.014	0.090^a^ ± 0.018	0.085^a^ ± 0.023	0.0232	-0.02	0.05
GR1012	0.065 ± 0.015	0.059 ± 0.014	0.055 ± 0.016	0.0888	0.01	-0.05
GR612	0.203^b^ ± 0.023	0.219^a^ ± 0.017	0.209^ab^ ± 0.015	0.0085	-0.01	0.06

BW6: 6-week body weight, BW8: 8-week body weight, BW10: 10-week body weight, BW12: 12-week body weight, BWG68: body weight gain from 6 to 8 weeks of age, BWG810: body weight gain from 8 to 10 weeks of age, BWG1012: body weight gain from 10 to 12 weeks of age, BWG612: body weight gain from 6 to 12 weeks of age, ADG: average daily weight gain, GR68: growth rate from 6 to 8 weeks of age, GR810: growth rate from 8 to 10 weeks of age, GR1012: growth rate from 10 to 12 weeks of age, GR612: growth rate from 6 to 12 weeks of age

The data presented in Table 4 showed that the three rabbit breeds’ growth traits are influenced by the SNP c.195 C > T in the *PGAM2* gene. The homozygous TT and heterozygous CT genotypes had higher BW12, BWG612, and ADG than the CC genotype in the NZW rabbits, with a significant additive and dominance effects for BW12, and BWG612 and a significant negative additive effect for BW6. The heterozygous genotype CT in the V-line rabbits, however, showed higher values for BW10 and BW12 compared with the CC genotype, with significant additive (BW10, BW12, and BWG612) and dominance effects (BW12 and BWG612) effects. In BR individuals, CC genotype rabbits had lower BWG1012, BW612, and ADG values than the CT rabbits, and had lower BWG810 than the TT genotype, with highly significant additive (BW12 and BWG810) and dominance (BW12 and BWG612) effects.

**Table 4:** Association between genotypes of the c.195 C > T mutation of the *PGAM2* gene and growth traits (Mean ± SD) in different rabbit breeds

	Genotype			*p*-value	Additive	Dominance
Trait	CC	CT	TT			
	New Zealand White rabbits					
BW6	857.73 ± 92.40	852.72 ± 92.59	829.33 ± 69.28	0.4729	-28.40*	9.19
BW8	1219.64 ± 126.27	1242.15 ± 85.44	1230.00 ± 70.28	0.6352	10.36	17.33
BW10	1807.55 ± 129.34	1848.22 ± 89.53	1874.33 ± 103.42	0.0952	66.78*	7.28
BW12	2280.73^b^ ± 134.53	2358.87^a^ ± 117.35	2358.17^a^ ± 137.88	0.0478	77.44**	39.42*
BWG68	361.91 ± 108.56	389.43 ± 81.31	400.67 ± 55.56	0.2686	38.76	8.14
BWG810	587.91 ± 135.04	606.07 ± 95.66	644.33 ± 107.5	0.1967	56.42	-10.05
BWG1012	473.18 ± 134.44	510.65 ± 94	483.83 ± 120.3	0.3752	10.65	32.15*
BWG612	1423.00^b^ ± 118.59	1506.15^a^ ± 131.21	1528.83^a^ ± 127.15	0.0135	105.83**	30.24*
ADG	33.88^b^ ± 2.824	35.86^a^ ± 3.12	36.40^a^ ± 3.03	0.0135	2.52	0.72
GR68	0.087 ± 0.026	0.093 ± 0.021	0.099 ± 0.015	0.1704	0.012	0.000
GR810	0.097 ± 0.025	0.099 ± 0.016	0.103 ± 0.017	0.4756	0.006	-0.001
GR1012	0.058 ± 0.017	0.061 ± 0.012	0.058 ± 0.013	0.5484	0.000	0.003
GR612	0.228 ± 0.02	0.234 ± 0.022	0.241 ± 0.016	0.0937	0.013	0.000
	**Baladi Rabbits**					
BW6	659.71 ± 88.61	645.28 ± 82.27	643 ± 54.55	0.7759	-16.71	-6.08
BW8	915 ± 71.03	909.52 ± 99.67	857.64 ± 123.18	0.2260	-57.36	23.20
BW10	1472.13 ± 160.43	1424.12 ± 164.84	1519.36 ± 220.24	0.3008	47.23	-71.63
BW12	1861.33 ± 175.33	2004.12 ± 250.1	1940.45 ± 154.45	0.0625	79.12*	103.23**
BWG68	255.29 ± 85.17	264.24 ± 96.98	214.64 ± 108.05	0.3455	-40.65	29.28
BWG810	557.13^b^ ± 134.48	514.60^b^ ± 104.7	661.73^a^ ± 206.16	0.0187	104.60*	-94.83
BWG1012	389.21^b^ ± 141.73	580.00^a^ ± 171.99	421.09^b^ ± 191.35	0.0004	31.88	174.85
BWG612	1201.63^b^ ± 128.96	1358.84^a^ ± 227.76	1297.45^ab^ ± 148.38	0.0129	95.82	109.30**
ADG	28.61^b^ ± 3.07	32.35^a^ ± 5.42	30.89^ab^ ± 3.53	0.0128	2.28	2.60
GR68	0.083 ± 0.03	0.084 ± 0.031	0.071 ± 0.03	0.4728	-0.012	0.007
GR810	0.116^b^ ± 0.023	0.111^b^ ± 0.018	0.137^a^ ± 0.036	0.0142	0.021	-0.016
GR1012	0.058^b^ ± 0.021	0.084^a^ ± 0.023	0.062^b^ ± 0.032	0.0012	0.004	0.024
GR612	0.239^b^ ± 0.018	0.256^a^ ± 0.027	0.249^ab^ ± 0.020	0.0386	0.010	0.012
	**V-line Rabbits**					
BW6	930.71 ± 68.44	956.48 ± 73.18	974.13 ± 144.39	0.4345	43.42	4.06
BW8	1280.14 ± 131.75	1332.91 ± 97.41	1322.88 ± 79.41	0.2382	42.74	31.40
BW10	1772.29^b^ ± 94.3	1902.22^a^ ± 146.57	1816.25^ab^ ± 163.73	0.0064	43.96**	107.95**
BW12	2270.79^b^ ± 67.13	2379.61^a^ ± 113.99	2360.19^a^ ± 106.47	0.0049	89.40*	64.12
BWG68	349.43 ± 93.4	376.43 ± 81.9	348.75 ± 128.11	0.4756	-0.68	27.34
BWG810	492.14 ± 82.5	569.3 ± 125.61	493.38 ± 158.64	0.0525	1.24	76.54
BWG1012	498.5 ± 93.89	477.39 ± 112.3	543.94 ± 96.55	0.1033	45.44	-43.83
BWG612	1340.07 ± 65.09	1423.13 ± 111.58	1386.06 ± 172.46	0.0752	45.99*	60.07*
ADG	31.91 ± 1.55	33.88 ± 2.66	33.00 ± 4.11	0.0751	1.09	1.43
GR68	0.078 ± 0.018	0.083 ± 0.017	0.078 ± 0.031	0.6416	0.000	0.005
GR810	0.081 ± 0.018	0.089 ± 0.018	0.077 ± 0.024	0.0865	-0.004	0.010
GR1012	0.063^ab^ ± 0.013	0.056^b^ ± 0.015	0.066^a^ ± 0.015	0.0341	0.003	-0.009
GR612	0.21 ± 0.014	0.214 ± 0.016	0.209 ± 0.030	0.5679	-0.001	0.005

BW6: 6-week body weight, BW8: 8-week body weight, BW10: 10-week body weight, BW12: 12-week body weight, BWG68: body weight gain from 6 to 8 weeks of age, BWG810: body weight gain from 8 to 10 weeks of age, BWG1012: body weight gain from 10 to 12 weeks of age, BWG612: body weight gain from 6 to 12 weeks of age, ADG: average daily weight gain, GR68: growth rate from 6 to 8 weeks of age, GR810: growth rate from 8 to 10 weeks of age, GR1012: growth rate from 10 to 12 weeks of age, GR612: growth rate from 6 to 12 weeks of age

## Discussion

Genetic improvement of rabbits using qualitative genetics approaches was successful and resulted in the development of new breeds and lines (Khalil and Bolet 2010). Nevertheless, there is a need for new approaches to speed up the selection process with more accuracy and to overcome the limitations of traditional selection. Marker-assisted selection (MAS) offers an accurate, reliable, and low-cost approach that can be used for the genetic improvement of local breeds that exist in limited numbers. When the marker effect is known for a given trait, animals can be evaluated without a phenotype based on the existence of markers (Meuwissen and Goddard 1996; Helal and El-Gendy 2023). This strategy can increase the rate of genetic gain (De Roos et al. 2011; Helal et al. 2024), and cut the cost of breeding schemes by approximately 92% (Schaeffer 2006). Moreover, it is very useful in the improvement of traits that are difficult to measure in live animals such as carcass traits, and traits that are expressed in a single sex, such as reproductive traits. Additionally, in the case of expensive phenotypic recording for numerous economic traits, the use of genotype information as a selection aid can be a reasonable alternative (Yang et al. 2013).

One of the main requirements for MAS practice is the estimation of the effect of different markers on the traits of interest. The estimations of SNP effects can theoretically be applied for a few generations with just a minimal loss in prediction accuracy; hence, it is not required to test the crossbred phenotypes every generation (Szkiba et al. 2014). In this regard, there are some recent efforts to estimate the effect of different SNPs of different candidate genes that affect the important traits in rabbits (Fontanesi 2016; Safaa et al. 2023).

The goal of this research was to identify DNA markers whose variability may be related to rapid growth features using the candidate gene technique in portions of two growth-related genes (*NPY* and *PGAM2*) that are located on the same chromosome. PCR-Csp6I digestion of 855 kb was used for genotyping the *PGAM2* gene and HRM was used for genotyping the g.1778G > C SNP. The three genotypes for the two investigated genes were detected. The different genotypes were distributed differently among the three rabbit populations. All genotypic frequencies followed the Hardy-Weinberg distribution, indicating the goodness of animal sampling.

The results revealed significant associations between *NPY* and *PGAM2* genotypes and growth traits at different levels in the three breeds. The polymorphisms found in the three breeds of high growth-rate rabbits may modify the activity or functionality of the *NPY* and *PGAM2* genes in rabbits. To the best of our knowledge, there is very little information about investigating polymorphisms in the *NPY* and *PGAM2* genes and their association with growth traits in rabbits, as reported previously (Khalil 2020; Helal et al. 2022). The two studied SNPs showed high PIC and heterozygosity values, indicating high levels of genetic variation, and therefore, a high genetic progress through selection is expected. Additionally, many traits associated with the SNPs of the two genes showed significant additive and dominance variance that could be consumed by breeding programs. The observed oscillations in the additive and dominance effect may be attributed to two main reasons, the first reason is the pleiotropic effect of the two genes, where the relative contribution may change with age, leading to oscillations in the observed genetic effects (Charmantier et al. 2006). The second reason is that the same allele may have an opposite effects on the same trait across ages (Maklakov et al. 2015; Everman and Morgan 2018).

Liu et al. (2014) sequenced the *NPY* gene in Ira, Champagne, and Tianfu black rabbits. According to the findings, intron 1 of the rabbit *NPY* gene contains four SNPs, namely, g.1491G > A, g.1525G > T, g.1530 C > T, and g.1778G > C. The g.1778G > C SNP was genotyped further, but it was not significantly associated with body weight at different ages. However, the eviscerated slaughter % and semi-eviscerated slaughter percentage were substantially connected with that SNP, and those with the CC genotype performed better than those with the CG genotype. The results of the current study, on the contrary, indicated that allele G is preferred in growth traits over allele C, and GG genotype rabbits had better performance than the CC genotype. These differences in the results of our study and previous work may be attributed to the genetic background differences between the studied animals (Ateya et al. 2021). Furthermore, the results of the current study also indicated that this SNP seems to affect late growth traits rather than post-weaning growth traits, and therefore, it is more likely to affect carcass traits.

The link between the *NPY* gene and growth performance has been addressed in different farm animals. In beef cattle, three SNPs in intron 2 of the *NPY* gene were strongly correlated with body weight, ADG, feed conversion ratio (FCR), and marbling (Sherman et al. 2008). In Chinese indigenous cattle, polymorphisms of the *NPY* gene also significantly affected body length and chest girth (Zhang et al. 2011). We hypothesized that *NPY* may also be a candidate gene and relevant given the substantial conservation of the NPY protein structure and DNA sequence in rabbits and other livestock.

Ateya et al. (2021) characterized SNPs of the *PGAM2* gene in three breeds of rabbits (V-line, Baladi Black, and Baladi Red rabbits) concerning the association of *PGAM2* gene polymorphisms in rabbits. The nucleotide sequence alignment revealed no differences between the sequences of the tested breeds, indicating that the *PGAM2* gene is highly conserved in rabbits. Genetic polymorphisms were examined on various *PGAM2* gene fragments, which may explain the differences between our study and earlier research. Additionally, Wu et al. (Wu et al. 2015) sequenced part of the *PGAM2* gene in Tianfu black, Ira, and Champagne rabbit breeds, and detected three SNPs. c.195 C > T was genotyped further to investigate the association between growth traits, and the heterozygous (CT) genotype of c.195 C > T was associated with body weight at 84 days of age and with average daily weight gain. These results were in agreement with the results obtained in the current study, where the SNP c.195 C > T significantly affected body weight at 10 and 12 weeks of age, and did not affect the post-weaning weights. Additionally, the results indicated the advantage of the mutant allele T, in favor of the heterozygous genotype in BR and V-line rabbits. In contrast, contrary, the same mutation of the *PGAM2* gene was genotyped in V-line, Alexandria rabbits (El-Sabrout and Aggag 2017), and in meat line P91 rabbits (Nahácky et al. 2018), but the association with growth traits was insignificant. In ducks, *PGAM2* was considered a candidate gene for the rigorous selection of growth performance (Ghanem et al. 2023).

In conclusion, the results in the present study revealed the evaluation of the genetic variations in the *NPY* and *PGAM2* genes among NZW, BR, and V-line rabbit breeds. The two SNPs were significantly correlated with growth traits in the different breeds, with special emphasis on late growth traits. The *NPY* and *PGAM2* genes may therefore serve as candidates and proxies for indicators of rabbit growth features.

## Data Availability

Data available upon request from the authors.
